# Corn-dependent exercise-induced anaphylaxis

**DOI:** 10.1186/1710-1492-10-S1-A26

**Published:** 2014-03-03

**Authors:** Mitra Abaeian, Rozita Borici-Mazi

**Affiliations:** 1 Department of Medicine, Queen’s University, Kingston ON, Canada

## Background

Exercise-induced anaphylaxis is a rare disorder characterized by the development of a severe allergic response occurring after mild- to-strenuous physical activity. Food-dependent exercise-induced anaphylaxis (FDEIA) comprises 30-50% of all cases of exercise-induced anaphylaxis [2]. Diagnosis involves careful history and appropriate skin prick testing and food specific IgE levels [4,6]. However, food dependent exercise challenges may be required to confirm the diagnosis [6]. We describe the case of a 51 year old male, diagnosed with corn dependent exercise anaphylaxis via serial exercise challenges.

## Methods

Case report and literature review.

## Results

A 51 year old male military pilot described the following symptoms 10 minutes after running on the treadmill: sudden onset palm pruritus, bilateral arm tingling, urticarial rash on his arms and chest, tongue and eyelid swelling, and difficulty speaking/swallowing. He denied wheezing, shortness of breath, gastrointestinal symptoms, or light-headedness. He was self-treated with 50 mg of diphenhydramine and loratadine. His symptoms resolved in 2-3 hours. He had consumed a Shepherd’s Pie approximately 45 minutes prior to exercise. The ingredients included beef, potato, corn, wheat, flour, and Worchestershire sauce. He denied ingestion of ASA/NSAIDS or alcoholic beverages prior to the event. His past medical history was remarkable for hypercholesterolemia and diet-controlled Diabetes Mellitus. He had a history of seasonal allergies for which he was on maintenance allergen immunotherapy. His medications included Crestor, Coenzyme Q10, and a fiber supplement. Physical examination was unremarkable. Skin prick testing to the individual ingredients of the Shepherd’s Pie were negative. IgE levels were positive for corn and wheat, and negative to the other ingredients. Patient was advised a wheat and corn free diet and was able to resume his exercise routine. Subsequently, he underwent serial food dependent exercise challenges. The exercise challenges verified that corn was the culprit food. The patient was advised to stop eating corn, introduce wheat, and resume his regular exercise routine. He had two minor episodes of urticaria with exertion in the following year, confirmed to be from accidental exposure to corn.

## Conclusions

This case demonstrated that, although wheat is one of the most common triggers of food dependent exercise induced anaphylaxis, corn was the culprit for this patient’s exercise induced symptoms. Food dependent exercise challenges can be time consuming and associated with risks, but yet important to verify the diagnosis when multiple triggers are suspected.
